# High Mass Accuracy Phosphopeptide Identification Using Tandem Mass Spectra

**DOI:** 10.1155/2012/104681

**Published:** 2012-07-15

**Authors:** Rovshan G. Sadygov

**Affiliations:** Sealy Center for Molecular Medicine, Department of Biochemistry and Molecular Biology, The University of Texas Medical Branch, Galveston, TX 77555, USA

## Abstract

Phosphoproteomics is a powerful analytical platform for identification and quantification of phosphorylated peptides and assignment of phosphorylation sites. Bioinformatics tools to identify phosphorylated peptides from their tandem mass spectra and protein sequence databases are important part of phosphoproteomics. In this work, we discuss general informatics aspects of mass-spectrometry-based phosphoproteomics. Some of the specifics of phosphopeptide identifications stem from the labile nature of phosphor groups and expanded peptide search space. Allowing for modifications of Ser, Thr, and Tyr residues exponentially increases effective database size. High mass resolution and accuracy measurements of precursor mass-to-charge ratios help to restrict the search space of candidate peptide sequences. The higher-order fragmentations of neutral loss ions enhance the fragment ion mass spectra of phosphorylated peptides. We show an example of a phosphopeptide identification where accounting for fragmentation from neutral loss species improves the identification scores in a database search algorithm by 50%.

## 1. Introduction

The reversible phosphorylation of proteins regulates many aspects of cell life [1–3]. Phosphorylation and dephosphorylation, catalyzed by protein kinases and protein phosphatases, can change the function of a protein, for example, increase or decrease its biological activity, stabilize it or mark it for destruction, facilitate or inhibit movement between subcellular compartments, initiate or disrupt protein-protein interactions [1]. It is estimated that 30% of all cellular proteins are phosphorylated on at least one residue [4]. Abnormal phosphorylation is now recognized as a cause or consequence of many human diseases. Several natural toxins and tumor promoters produce their effects by targeting particular protein kinases [5, 6] and phosphatases. Protein kinases catalyze the transfer of the *γ*-phosphate from ATP to specific amino acids in proteins; in eukaryotes, these are usually Ser, Thr, and Tyr residues.

**Figure 1:**
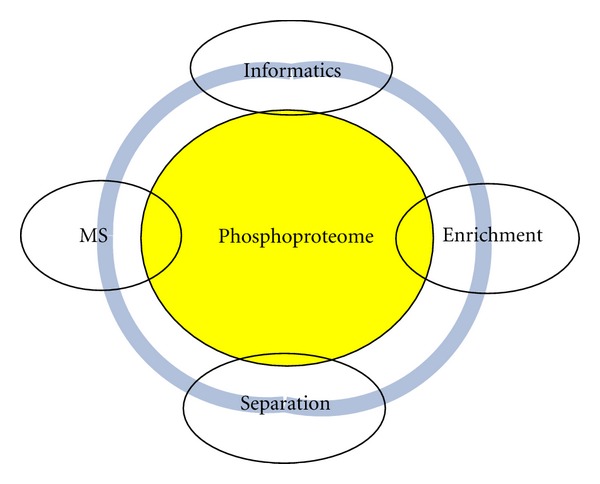
Phosphoproteomics and its constituent parts.

Mass-spectrometry-based proteomics has emerged as a powerful platform for the analysis of protein phosphorylations [7]. In particular, the shotgun proteomics [8], using liquid chromatography coupled with mass spectrometry (LC-MS), has been successfully employed for comprehensive analysis of global phosphoproteome [6, 9, 10]. The advances in the phosphoproteomics were driven by developments in mass spectrometry (high resolution and mass accuracy), peptide/protein separation, phosphopeptide/protein enrichment, peptide fragmentation [11, 12], quantification, and bioinformatics data processing, Figure 1. Currently, thousands of the phosphopeptides can be detected and quantified in just one experiment. Excellent recent reviews describe experimental procedures involved in phosphoproteomics [13, 14]. Bioinformatics processing is recognized as an integral part of phosphoproteome analysis. Several applications have been developed for phosphopeptide identifications [15, 16], phosphorylation site localization [17, 18], and quantification [19]. Tandem mass spectra are searched for phosphopeptides from protein sequences with potential modifications on Ser, Thr, and Tyr residues. The searches are not targeted. Every modifiable residues can be either modified or unmodified. The effective peptide search space increases exponentially leading to computational complexity as well as possible false identifications. High mass accuracy afforded by the modern mass spectrometers enables reducing the complexity of the search space by applying tighter bounds on peptide masses.

Lu and coworkers [20, 21] have developed models based on support vector machine (SVM) to screen for phosphopeptide spectra and validate their identifications. Their approach accurately explains spectra from phosphorylated peptides. However, SVM also acts like a black box, and it is difficult to gain insights into specifics of its decision making. Another development had used dynamic programming to relate spectra of modified and unmodified forms of a peptide [22]. This approach identifies modified peptides by comparing their tandem mass spectra with the annotated tandem mass spectra of unmodified peptides. The search space is restricted to peptides positively identified in unmodified form.

Here, we describe the informatics aspects of phosphopeptide identifications using protein sequence databases and mass spectral data from high mass accuracy and resolution instruments. Database identifications of phosphorylated peptides are done in a dynamic mode—assuming that in a peptide sequence Ser, Thr, and Tyr may or may not be are modified. For database searches, it effectively means exponential increase in the size of database. About 17% of amino acid residues (of which Ser 8.5%, Thr 5.7%, Tyr 3.0%) [23] in human proteome can potentially be phosphorylated. In general, if there are N amino acid residues which can potentially be phosphorylated, the effective database size could increase by as much as 2^N^ times.

## 2. Informatics Aspects of the**** Phosphoproteomics

### 2.1. Spectra Extraction

LTQ-Orbitrap mass spectrometer [24] stores the mass spectra in a proprietary “raw” file format (ThermoFisher Scientific, San Jose, CA). extract_msn algorithm extracts spectral information from the raw file and converts it into text file format for further data processing. It uses a built-in module to evaluate isotopic envelope of mass species. From the isotope distribution, extract_msn determines the monoisotopic mass and charge state of a peptide. Both of these are critically important and used by database search algorithms.

**Figure 2:**
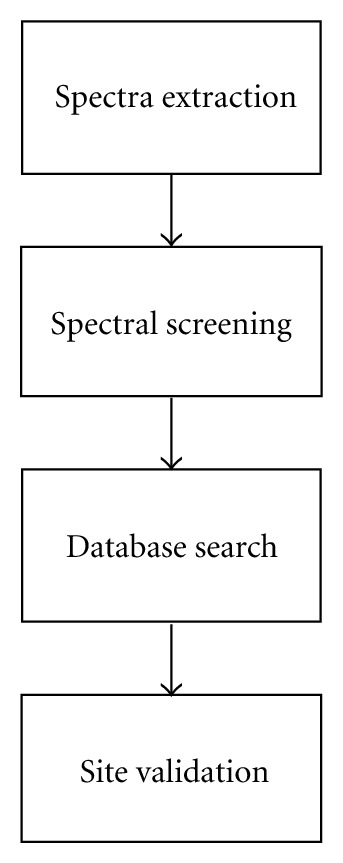
General informatics flowchart of a phosphoproteomics analysis.

Normally, the full MS scan is recorded in the Orbitrap mass analyzer which is a high resolution and mass accuracy mass analyzer. The routine mass accuracy of intact peptides is in the range of ±5–10 part-per-million (ppm). This is a very high mass accuracy and is very important for reducing false discovery rates of peptide identifications. The accuracy of the intact peptide's mass affects the number of candidate peptides from the database that will be considered in matching to the spectra. The candidate peptides are filtered based on the mass of the intact peptide and accuracy with which the mass has been measured. The higher the accuracy, the smaller the number of candidate peptides, and as a result the smaller the possibility of false positives. Fragment ion masses are recorded in ion trap mass analyzer. This is a very sensitive mass analyzer. However, the mass accuracy of measured ions is nominal, and normally in the range of ±0.5 Da.  Figure 2 summarizes the informatics flowchart of a phosphoproteomics analysis.

### 2.2. Database Searching

Peptide identification using tandem mass spectra and protein databases is an integral part of proteomics. It is important that peptide assignments are determined with high accuracy and are verifiable. In high-throughput experiments, when thousands of tandem mass spectra are searched, it is not practical for an expert user to manually assign every spectrum and the assignments are made by software. The software uses a concept, either heuristic or probabilistic model, to measure similarity between experimental tandem mass spectrum and an amino acid sequence. For high quality spectra, when signal-to-noise ratio is high and spectra contains clearly defined ion series, most of the programs and concepts perform very well. However, when the peptide fragmentation is poor and the spectrum contains very few distinguishable peaks, or if the peptide amino acid sequence is not in the database, a number of factors lead to a wrong peptide assignment—a false identification. Depending on the software, sometimes false identifications may yield high assignment scores. High mass accuracy may substantially reduce the false identification by restricting the search space of allowed candidate sequences.

Another important aspect of the database search algorithm is the modeling of the fragmentation pattern. This is especially true for cases when there is significant difference from the routine fragmentation pattern, caused, for example, by posttranslational modifications. Thus, it is known that, in CID, peptides containing phosphorylated amino acid residues, Ser or Thr, tend to lose the phosphor group(s) before they fragment along the peptide backbone. We show here that accounting for product ions in this pathway significantly improves the identification scores of phosphopeptides in SEQUEST database search algorithm.

### 2.3. Assignment of Phosphorylation Sites

It is often difficult to differentiate between possible phosphorylation sites in a peptide and to uniquely assign phosphorylated amino acid residues. It has to do with the presence of several Ser, Thr, and Tyr residues in a peptide and low overall intensity of peaks from product ions of phosphorylated peptides. To address this problem, Beausoleil and coworkers [17] have developed a probability-based approach to determine phosphorylation sites of peptides from the results of SEQUEST database search algorithm [16]. Their model divides the spectra into the mass intervals of equal width. In every interval, only 6 to 8 (dependent on the intensity) peaks are retained and the rest of the peaks are ignored. In matching to the experimental peaks, only the modified fragments are considered. The probability of phosphorylation site determination is estimated via a binomial probability. This model has been successful in many practical applications especially for high scoring peptides.

## 3. Discussions

There are several mass spectral characteristics of phosphorylated peptides. Thus, in collision-induced dissociation reactions, one of the most prevalent pathways of the molecular dissociation is the neutral loss of the labile phosphor group of Ser or Thr residues. The presence of the relevant ion often serves as a diagnostic feature for phosphorylated peptides [25]. Often the product ions in spectra include two ion series, one from the phosphorylated peptide and the other from the precursor peptide that has lost the phosphor group(s). We have previously modified SEQUEST database search algorithm to account for the two ion sequences when identifying phosphorylated peptides [26]. The development has helped to improve the sensitivity of the phosphor peptide identifications and location of phosphorylation sites. Here, we demonstrate, in the example of this algorithm, the advantages of using extensive fragmentation pathways for targeted analysis.

In general, fragmentation of peptides via CID produces strong b- and y-ion series [27]. Therefore, most database search algorithms generate b- and y-ions (and corresponding water and ammonia losses from them) for the theoretical spectrum of a candidate amino acid sequence. In the tandem mass spectra of phosphorylated peptides, additional fragmentation patterns are observed. In addition to the b- and y-ions of the original phosphorylated peptide, ions that originate from phosphor group losses, 98 Da (from Ser or Thr residues), are also present. We accounted for these fragments by augmenting the fragmentation pattern correspondingly to add neutral loss fragments from the phosphorylated amino acid residues. The fragmentation model [28] was used for both, preliminary and cross-correlation scores in SEQUEST. Cross-correlation scores of phosphorylated peptides generated from new fragmentation pattern were about 50% higher.

**Figure 3:**
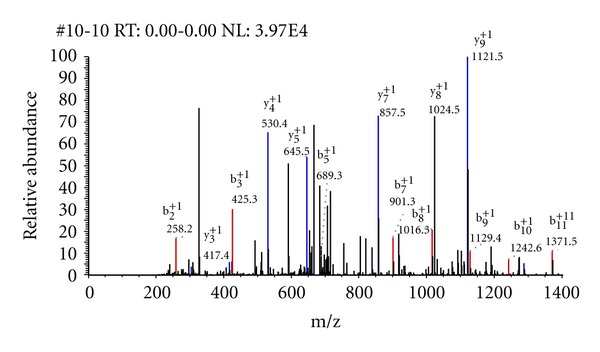
An example spectrum of phosphorylated peptide, R.TRS*PS*PDDILER.V. The cross-correlation scores before and after SEQUEST fragment ion modifications are 4.97 and 3.14, respectively.

The interpretations of the tandem mass spectra of phosphorylated peptides may be complicated. The main reason for this is the low abundance of fragment ions due to the alternative fragmentations. One experimental approach used for enhancing phosphopeptide identifications is to do a higher-order mass spectrometry on the fragment ions of original precursor. In these experiments, neutral loss fragment ions generated during CID in MS^2^ are further dissociated generating MS^3^ spectra. In spectra collected with this approach, there are number of ions corresponding to phosphoric acid group losses from b- and y-ions. An example of such a tandem mass spectrum is shown in Figure 3. SEQUEST matched this spectrum to the phosphorylated peptide sequence, R.TRS*PS*PDDILER.V. The cross-correlation and preliminary scores in the model not including phosphoric acid loss fragmentations were 3.14 and 1029.6, respectively. When we included the neutral loss ions, the cross-correlation and preliminary scores were 4.97 and 2353.6, respectively. The total number of theoretical ions generated for this peptide was 44. 35 of these ions matched to product ions in the spectrum. In contrast, 16 of the 22 theoretical ions matched the tandem mass spectrum in the original model (ignoring neutral loss fragments). The results on this and other phosphorylated peptide spectra showed that a realistic model of product ions of phosphorylated peptides needs to account for the fragments resulting from neutral (phosphoric acid group) loss of the b- and y-ions. The procedure has been automated and is used in the case of dedicated MS^n^ experiments to enhance fragmentation spectra of phosphopeptides.

## 4. Conclusion

Increased mass accuracy for precursor ions combined with enhanced fragmentation pathways helps bioinformatics methods to improve phosphopeptide identifications from tandem mass spectra and protein sequence databases. Normally identifications of phosphorylated peptides yield small cross-correlation scores. This has partially to do with the theoretical fragmentation models, which take into account only b- and y-ions generated from the peptide bond fragmentations of phosphorylated precursor peptides. We augmented the fragmentation pattern (in SEQUEST) [26] introducing theoretical peaks for b- and y-ions from neutral loss precursors and fragments. Cross-correlation scores of phosphorylated peptides increased by up to 50% using the enhanced fragmentation model.

## Abbreviations

Da: Dalton
ppm: Parts per million
Ser: Serine
Thr: Threonine
Tyr: Tyrosine
CID: Collision induced dissociation
LC: Liquid chromatography
MS: Mass spectrometry.

## References

[1] Cohen P (2002). The origins of protein phosphorylation. *Nature Cell Biology*.

[2] Ubersax JA, Ferrell JE (2007). Mechanisms of specificity in protein phosphorylation. *Nature Reviews Molecular Cell Biology*.

[3] Manning G, Whyte DB, Martinez R, Hunter T, Sudarsanam S (2002). The protein kinase complement of the human genome. *Science*.

[4] Cohen P (2000). The regulation of protein function by multisite phosphorylation—a 25 year update. *Trends in Biochemical Sciences*.

[5] Andersen JN, Sathyanarayanan S, Di Bacco A (2010). Pathway-based identification of biomarkers for targeted therapeutics: personalized oncology with PI3K pathway inhibitors. *Science Translational Medicine*.

[6] Moritz A, Li Y, Guo A (2010). Akt-RSK-S6 kinase signaling networks activated by oncogenic receptor tyrosine kinases. *Science Signaling*.

[7] Zhou H, Watts JD, Aebersold R (2001). A systematic approach to the analysis of protein phosphorylation. *Nature Biotechnology*.

[8] Link AJ, Eng J, Schieltz DM (1999). Direct analysis of protein complexes using mass spectrometry. *Nature Biotechnology*.

[9] Huttlin EL, Jedrychowski MP, Elias JE (2010). A tissue-specific atlas of mouse protein phosphorylation and expression. *Cell*.

[10] Zhai B, Beausoleil SA, Mintseris J, Gygi SP (2008). Phosphoproteome analysis of Drosophila melanogaster embryos. *Journal of Proteome Research*.

[11] Coon JJ, Ueberheide B, Syka JEP (2005). Protein identification using sequential ion/ion reactions and tandem mass spectrometry. *Proceedings of the National Academy of Sciences of the United States of America*.

[12] Jedrychowski MP, Huttlin EL, Haas W, Sowa ME, Rad R, Gygi SP (2011). Evaluation of HCD- and CID-type fragmentation within their respective detection platforms for murine phosphoproteomics. *Molecular & Cellular Proteomics*.

[13] Wang F, Song C, Cheng K, Jiang X, Ye M, Zou H (2011). Perspectives of comprehensive phosphoproteome analysis using shotgun strategy. *Analytical Chemistry*.

[14] Nilsson CL (2012). Advances in quantitative phosphoproteomics. *Analytical Chemistry*.

[15] Ruttenberg BE, Pisitkun T, Knepper MA, Hoffert JD (2008). PhosphoScore: an open-source phosphorylation site assignment Tool for MS^n^ data. *Journal of Proteome Research*.

[16] Eng JK, McCormack AL, Yates JR (1994). An approach to correlate tandem mass spectral data of peptides with amino acid sequences in a protein database. *Journal of the American Society for Mass Spectrometry*.

[17] Beausoleil SA, Villén J, Gerber SA, Rush J, Gygi SP (2006). A probability-based approach for high-throughput protein phosphorylation analysis and site localization. *Nature Biotechnology*.

[18] Taus T, Kocher T, Pichler P (2011). Universal and confident phosphorylation site localization using phosphoRS. *Journal of Proteome Research*.

[19] Cox J, Matic I, Hilger M (2009). A practical guide to the MaxQuant computational platform for SILAC-based quantitative proteomics. *Nature protocols*.

[20] Lu B, Ruse C, Xu T, Park SK, Yates J (2007). Automatic validation of phosphopeptide identifications from tandem mass spectra. *Analytical Chemistry*.

[21] Lu B, Ruse CI, Yates JR (2008). Colander: a probability-based support vector machine algorithm for automatic screening for CID spectra of phosphopeptides prior to database search. *Journal of Proteome Research*.

[22] Tsur D, Tanner S, Zandi E, Bafna V, Pevzner PA (2005). Identification of post-translational modifications by blind search of mass spectra. *Nature Biotechnology*.

[23] Echols N, Harrison P, Balasubramanian S (2002). Comprehensive analysis of amino acid and nucleotide composition in eukaryotic genomes, comparing genes and pseudogenes. *Nucleic Acids Research*.

[24] Hu Q, Noll RJ, Li H, Makarov A, Hardman M, Cooks RG (2005). The Orbitrap: a new mass spectrometer. *Journal of Mass Spectrometry*.

[25] Lu B, McClatchy DB, Jin YK, Yates JR (2008). Strategies for shotgun identification of integral membrane proteins by tandem mass spectrometry. *Proteomics*.

[26] Sadygov RG, Shofstahl J, Humer A Improvements to the database search algorithm SEQUEST for accurate mass support and improved phosphorylation searching.

[27] Paizs B, Suhai S (2005). Fragmentation pathways of protonated peptides. *Mass Spectrometry Reviews*.

[28] Sadygov RG, Maroto FM, Hühmer AFR (2006). ChromAlign: a two-step algorithmic procedure for time alignment of three-dimensional LC-MS chromatographic surfaces. *Analytical Chemistry*.

